# Gender differences in insulin and C-peptide concentrations at birth using cord blood collection

**DOI:** 10.1590/2359-3997000000148

**Published:** 2016-02-11

**Authors:** Mohammad Shafi Kuchay, Rattan P. Kudyar, Anil Gupta, Kamal Kishor Pandita, Mohammad Ashraf Ganie

**Affiliations:** 1 Division of Endocrinology and Diabetes Medanta, The Medicity Haryana India Division of Endocrinology and Diabetes, Medanta, The Medicity, Sector-38, Haryana, India; 2 Postgraduate Department of Internal Medicine Acharya Shri Chander College of Medical Sciences and Hospital Jammu J&K India Postgraduate Department of Internal Medicine, Acharya Shri Chander College of Medical Sciences and Hospital, Jammu, J&K, India; 3 Department of Endocrinology and Metabolism All India Institute of Medical Sciences New Delhi India Department of Endocrinology and Metabolism, All India Institute of Medical Sciences, New Delhi, India

**Keywords:** Insulin resistance, cord plasma insulin, cord plasma C-peptide, newborn babies

## Abstract

**Objective:**

: To study gender differences in insulin and C-peptide concentrations at birth using cord blood collection.

**Subjects and methods:**

This study was conducted in a maternity hospital, in Jammu province of Jammu and Kashmir, India. All women with pregnancy who were hospitalized for delivery were followed. All pregnant ladies who had no medical condition affecting insulin levels, as per history and routine antenatal blood testing, were included in the study. The test for cord plasma insulin and C-peptide was done in 60 (30 males) full-term (≥ 37 completed weeks) normal delivery babies within 4 hours of the collection of samples using the electro-chemiluminescence immunoassay (ECLIA) on Roche elecsys module immunoassay analyzer. Weight of the babies was taken immediately after birth using digital scales.

**Results:**

Cord plasma insulin and C-peptide measured in EDTA were compared between boys and girls and also related to birth weight. Girls were lighter (2,830 ± 37 *vs.* 3,236 ± 46 g; *p* = < 0.001) but had higher cord insulin (16.48 ± 4.88 *vs.* 10.53 ± 4.04 µU/mL;* p* = < 0.001), and C-peptide (2.47 ± 0.66 *vs*. 0.834 ± 0.26 ng/mL; *p =* < 0.001) concentrations than newborn boys.

**Conclusion:**

Female newborn babies have higher cord plasma insulin and C-peptide concentrations than male newborns, despite being smaller, suggesting intrinsic insulin resistance in girls.

## INTRODUCTION

A number of studies suggest that girls are more insulin resistant than boys. This has been shown by using intravenous glucose tolerance tests, fasting insulin measurements and euglycemic clamps in children from age 5 years through latechildhood, puberty and adolescence. Euglycemic clamp studies of 357 normal children (10-14 years of age) demonstrated significant differences in insulin resistance between boys and girls (1). Lindsay and cols. observed a gender difference in cord blood insulin, proinsulinand 32-33 split proinsulin levels. Concentrations of insulin propeptides were higher in female than male offspring (2). A study of 307 children (5 years of age) was unable to explain gender differences by lookingat differences in anthropometry and physical activity. They observed that girls at five are intrinsically more insulin resistant than boys (3). Ehtisham and cols. studied the ethnic differences in insulin resistance and body composition in United Kingdom adolescents. They found that type 2 diabetes mellitus (T2DM) is increasingly recognized in childhood,occurring more frequently in the South Asians and in girls (4). All the above studies measured surrogate markers of insulin resistance in childhood and demonstrated gender differences in insulin resistance. It has been argued that this gender difference in insulin resistance may be due to differences in the environment and physical activity in childhood. Shields and cols. were the first to measure cord blood insulin and insulin-like molecules, thereby eliminating the effect of environment and physical activity on insulin resistance. They demonstrated that cord blood insulin, total proinsulin and intact proinsulin concentrations were higher in girls despite being lighter than boys, indicating intrinsic insulin resistance (5). The differences in cord blood insulin and C-peptide levels have not been studied in Indian newborns. Jammu and Kashmir has an ethnic population with relatively similar genetics and environment. Therefore, this study was designed to demonstrate the differences in cord plasma insulin and C-peptide levels in normal newborn Indian babies, born of normal parents.

## SUBJECTS AND METHODS

This study was conducted in a maternity hospital in Jammu province of Jammu and Kashmir, India. All women with pregnancy who got admitted for delivery were considered for inclusion in the study. Women with pregnancy who had no history of T2DM, hypertension, smoking, obesity, liver diseases or other chronic diseases were followed till delivery. Exclusion of study subjects was done on the basis of history, anthropometry, and review of investigations done during the antenatal period. All pregnant subjects had liver function tests, renal function tests, complete blood counts, oral glucose tolerance test, ultrasonography for fetal well-being, tests for human immunodeficiency virus (HIV 1 and 2), hepatitis B (HbsAg) and hepatitis C (anti-HCV) done as routine in antenatal care clinic. Only newborn subjects whose parents were non-diabetic, non-obese; who had no liver disease or other conditions related to insulin resistance syndrome were included in the study. All the babies born by normal vaginal delivery and who had normal birth weight (≥ 2500 g to < 4000 g) were included in the study.

Informed consent was obtained from all the parents of the newborns; the study was approved by institutional ethics committee and was carried out in accordance with the principles of the Declaration of Helsinki.

A 5-mL sample of cord blood of sixty babies born by normal vaginal delivery was collected immediately following delivery of the placenta. This was transferred to potassium EDTA tubes. The samples were centrifuged and the plasma separated. The test for cord plasma insulin and C-peptide was done within 4 hours of the collection of samples using the electro-chemiluminescence immunoassay (ECLIA) on Roche elecsys module immunoassay analyzer. Weight of the babies was taken immediately after birth using digital scales.

The Statistical Package for Social Sciences (SPSS) version 21.0 was utilized to analyze the results. The independent student t was used to analyze the sample divided into two groups related to male and female babies. p-values < 0.05 were considered statistically significant. Fifty percent (n = 30) of the sample was male and fifty percent (n = 30) was female.

## RESULTS

Cord plasma from girls had higher concentrations of insulin (16.48 ± 4.88 *vs*. 10.53 ± 4.04 µU/mL; *p* = < 0.001), and C-peptide (2.47 ± 0.66 *vs*. 0.834 ± 0.26 ng/mL; *p =* < 0.001) than that of boys. Girls were lighter (2,830 ± 37 *vs*. 3,236 ± 46 g; *p* = < 0.001) than boys (Table 1). Gestational age had no correlation with weight of babies (r = -0.038, *p* = 0.077) or with insulin levels (r = -0.154, *p* = 0.240) or with C-peptide levels (r = -0.081, *p* = 0.537). Weight of babies was correlated with both insulin levels (r = 0.557, *p* = < 0.001) as well as C-peptide levels (r = 0.855, *p* = < 0.001) (Table 2).

**Table 1:** Comparison of gestational age, weight, cord plasma insulin and C-peptide between boys and girls (mean ± SD)

Parameters (units)	Boys (Mean ± SD)	Girls (Mean ± SD)	p value
Gestational age (weeks)	39.27 ± 1.68	39.00 ± 1.68	0.541
Weight (g)	3236 ± 46	2830 ± 37	< 0.001*
Insulin (µU/mL)	10.53 ± 4.04	16.48 ± 4.88	< 0.001*
C-Peptide (ng/mL)	0.83 ± 0.26	2.47 ± 0.66	< 0.001*

* *p* *value *< 0.05 statistically significant.

**Table 2:** Correlation between study parameters

Parameters	Correlation Coefficient^**#**^(r)	p value
Gestational age *vs*. weight of babies	-0.038	0.0772
Gestational age *vs*. C-peptide levels	-0.081	0.537
Gestational age *vs*. insulin levels	-0.0154	0.240
Weight (babies) *vs*. insulin levels	0.557	< 0.001*
Weight (babies) *vs.* C-peptide levels	0.855	< 0.001*

# Pearson correlation coefficient; * *p* value < 0.05 statistically significant.

## DISCUSSION

This study provides data showing that normal newborn girls have higher insulin and C-peptide concentrations in cord plasma than newborn boys despite weighing less at birth. As insulin is a principal growth factor in utero (6,7), the higher insulin coupled with reduced growth in newborn girls suggests that girls are more insulin resistant in utero. Research has suggested that in childhood, girls are more insulin resistant than boys with higher insulin concentrations and have more adipose tissue at 5 years of age. Authors suggest that these differences could not be explained by other known determinants of insulin concentrations (3). Our finding that insulin and C-peptide in cord blood are higher in girls is consistent with an intrinsic difference between the sexes, which is unlikely to be determined by environmental factors. Our results also demonstrate that this phenotype of higher insulin resistance in girls is present right from birth.

As girls are born smaller (lighter and shorter) (3), with greater skinfold thickness (8), and in the presence of higher insulin concentrations than boys, they are intrinsically more insulin resistant because the increase in cord plasma insulin is not associated with augmented growth. Similar difference was seen between Indian and United Kingdom babies, where Indian babies were smaller with more adipose tissue and had higher plasma insulin concentrations (8). North American youth (10-19 years) with T2DM were obese and had a family history of T2DM, and were more likely to be girls than boys (9). Sex-linked genes may account for the intrinsic gender difference observed. These genes may have an important impacton the development of insulin resistance and the disorders associated with it, like T2DM and metabolic syndrome. This may also help to explain the occurrence of sex-linked polycystic ovary syndrome and female preponderance of T2DMin children (2,9). Much research is need in this area to identify genes responsible for gender differences in insulin resistance.

The strength of our study is uniform ethnic study cohort. Another strength is inclusion of only normal pregnant ladies, who had no previously known medical condition or detected during antenatal period, in this study. Furthermore, we included only babies who had no detected medical condition intranatally or postnatally, so as to exclude any influence on insulin levels. One of the limitations of our study is the measurement of plasma insulin and C-peptide levels as markers of insulin resistance, which are only surrogate markers of insulin resistance. Another limitation is the lack of information on the C-peptide levels or other measurements that could suggest insulin resistance in the mothers of these babies.

## CONCLUSION

This study provides evidence from cord plasma insulin and C-peptide measurements that girls are intrinsically more insulin resistant than boys at birth. As insulin resistance is one of the pathophysiology for many common disorders including T2DM and polycystic ovary syndrome, much research is needed in this area to look for the genetic cause(s) of insulin resistance in newborn female babies.
